# The Loyalty of Tourism in Synagogues: The Special Case of the Synagogue of Córdoba

**DOI:** 10.3390/ijerph17124212

**Published:** 2020-06-12

**Authors:** Juan Antonio Jimber del Río, Ricardo David Hernández-Rojas, Virginia Navajas-Romero, Amalia Hidalgo-Fernández

**Affiliations:** 1 Agricultural Economics, Finance and Accounting, Universidad de Córdoba, Avda. de Medina Azahara 5, 14071 Cordoba, Spain; et2heror@uco.es (J.A.J.d.R.); jjimber@uco.es (R.D.H.-R.); 2 Management, Economic Applied and Stadistics, Universidad de Córdoba, Avda. de Medina Azahara 5, 14071 Cordoba, Spain; vnavajas@uco.es

**Keywords:** tourism, synagogues, satisfaction, loyalty, Jewish, local community

## Abstract

Tourism in synagogues is an activity which is increasing in popularity due to the current interest in cultural and patrimonial heritage. The synagogue in Córdoba has become an irreplaceable Jewish tourist destination in Spain due to its origins, construction, conservation, and recognition by international organizations. This article analyzes the loyalty of tourism in synagogues using variables such as satisfaction, loyalty and the expected and perceived value of the destination and the historical monument using a structural research equations methodology. No previous studies on tourism at synagogues using structural equations have been made. The data was obtained by means of a survey given to 350 tourists visiting this cultural heritage site. The results showed that there is strong loyalty to the heritage site as well as the destination.

## 1. Introduction

Synagogues are unique monuments that can turn the areas where they are situated into attractive areas for tourism due to the unique characteristics that they offer. In the cultural literature, synagogues are important heritage sites that unite key factors of society such as religion and culture. They are one of the most important and least studied fields of multidisciplinary research this decade [1]. Tourism at synagogues can be seen to be a very specific type of cultural tourism for Jewish heritage, which is included in heritage tourism [2]. This type of tourism has gained importance in recent years [3], and studies related to the diaspora can be found (for example, [4]). This new interest has influenced studies into the concept of legacy, such as Jews who travel to sites of the Holocaust ([5,6]). In Europe, Spain is a tourist destination related to this Jewish legacy (see [7,8]). The increase in the number of Jewish descendants who want to connect with their historical legacy presents a growing opportunity for tourism in Europe, and adequate marketing strategies for this type of heritage have consequently been proposed [9].

Córdoba is an important, historic city that attracts cultural tourists from all over the world and is in the process of specializing in niche markets, such as Jewish heritage tourism. The city wants to strengthen and diversify its position as an international destination for cultural tourism for minority groups. The synagogue in Córdoba is a one of the most important cultural, religious and architectural Jewish heritage sites in Spain [10]. It has been recognized as a monument of Cultural Interest and is also part of the Historic Centre of Córdoba, which has been a UNESCO World Heritage Site since 1994 and has become the most-visited Jewish monument in the city of Córdoba, having had a total of 555,325 visitors in 2019 [11]. The importance of this monument can be seen from the increase of more than 200,000 visitors to the monument in 2015. This represents an annual growth of 12.14% in 2015. In following years, the number of visitors continued to grow, with increases of more than 5% per year reaching a total of 617,200 visits [11]. This monument must be studied in order to promote and manage it effectively from an ethnical point of view. There are many different ways to manage the business aspects of this type of heritage site for minority groups. Each management style will have different perceptions, beliefs, and processes, which, if not chosen correctly, could lead to a mistaken business development plan and, in extreme cases, hinder or prevent plans for cultural tourism by minority communities [12].

The theoretical model used for this study is the American Customer Satisfaction Index (ACSI) [13]. This model uses regular surveys to measure the level of satisfaction of American customers with national products and services. Customer satisfaction is measured on a scale from zero to 10, and the results gathered can be used to compare the companies and organizations in any business sector. The model has been used to measure customer loyalty in different areas such as health [14], online shopping [15], or banking [16]. This model has been widely used to study the tourism sector [17,18].

The aim of this article is to contribute to the existing literature in two ways. The first is with a review of tourism in synagogues and secondly, in a more practical manner, by providing ideas about heritage site management and cultural tourism for minority groups. These ideas help to improve the information available about the complexity of Jewish heritage tourism by using a theoretical model with a case study which analyzes the loyalty of tourists from minority groups, as mistaken management and advertising choices could promote negative aspects such as commercial exploitation, creating hierarchies and misunderstandings [19]. However, this type of tourism also presents opportunities for economic diversity and historical investigation, while accepting cultural diversity and minority groups. The satisfaction of tourists and their opinions about the perceived value of the Synagogue and the destination can be used to make recommendations, which can be useful for the different public and private entities are interested in promoting and improving this type of tourism. The information is given after analyzing how different factors influence the management and promotion of cultural tourism. Previous studies into ethnic minorities exist [19,20,21] but there are few that study this topic in Spain [7,22] and none of these use the American Customer Satisfaction Index (ACSI). There are studies which relate heritage and tourism with authenticity and loyalty at a destination [23], motivation and satisfaction [24], perceived value and loyalty [25], experience, satisfaction and tourist behavior [26] and satisfaction and loyalty [27]. However, there are no studies which measure satisfaction, perceived value and real value with loyalty to a heritage site and a synagogue with the type of cultural tourists that are investigated in this paper. Different points of view, approaches and ideas have been presented for the management of cultural assets from a cultural perspective. However, an important factor must be kept in mind when managing and promoting this type of tourism. These monuments have historical Jewish traditions and heritage and this factor can increase the loyalty of tourists. Therefore, this article also contributes to the literature on the management of heritage sites, because the opinions and criteria of the community that built and managed the site in the past may not have been taken into account. This consideration can help to find the management models that achieve higher levels of loyalty for a historically Jewish heritage site. The following investigation question is a result of these considerations: Do the tourist activities available at the Synagogue make tourists feel loyal to the site, as defined in the ACSI model, which takes into account perceived quality, perceived value and satisfaction?

## 2. Synagogues and Heritage Tourism

Synagogue tourism implies the special interest of tourists in learning about and experiencing a particular type of heritage. This type of tourism is the result of ideas about inheritance and legacy, and the tourist’s feelings of belonging, nostalgia and identity [28,29,30,31]. This type of tourist has a special interest in learning and getting in touch with a particular type of heritage. A wide range of tourism opportunities exist in a variety of countries such as Spain, the UK, Ireland, Germany and China with products offering trips and events related to legacy or heritage, family history and the genealogical interests of the customers [4]. This type of tourist can be interested in an extremely wide range of places, which include heritage beyond museums and places of historical interest, visits to churches, old houses and rural landscapes or synagogues with which tourists feel a personal connection, and which are not necessarily conventional tourist attractions [32].

Historic synagogues are heritage sites and, as such, are a resource for tourism [33]. In general terms, the academic literature on synagogue tourism is very scarce. The first indications of tourism carried out in synagogues date from the Second World War in the Warsaw ghetto with the visits of the first German Nazi “tourists” [34,35]. Studies into this type of tourism are varied, ranging from historical perspectives [36], employment [37], or the management of Jewish heritage [38]. Studies on synagogues in Spain have focused on the historical and archaeological areas, as well as an artistic point of view [22]. The Gazette of the Royal Academy of History contains texts from the 19th century regarding the Jewish aljama of Solsona [39]. The first paper on the synagogue of Córdoba was written by Castilla [40] and reported on the research (in 1878) into the artistic elements in the synagogue [41]. There are also archeological studies [42] about the Synagogue of Castillo de Lorca (Murcia). In addition to the studies mentioned from the 19th century, there have been others about the architectural intervention in the Synagogue of Valencia de Alcántara (Cáceres) [43]. Other researchers [44] have investigated the design and buildings of the network of Jewish Quarters in Spain for tourism purposes. The Jewish quarters of 24 cities were investigated along with the main monuments there. Not all the monuments are necessarily related to the Jewish world, but merely share the history and culture of the setting. There are also links to Jewish-inspired foods. It is possible to visit wineries where Kosher wine is produced and sold. The elements which have the biggest impact on tourist satisfaction are studied for both heritage and gastronomic tourism [45].

Heritage is usually classified and managed according to the meaning and value it is given locally and globally [46,47]. Heritage management and tourism management methods can come into conflict because of different factors, such as religion, culture and politics, which can cause the division and segmentation of the communities involved [48].

Studies into cultural tourism and heritage management analyze the impact that cultural diversity at tourist destinations can have on the evolution of tourism in different places [49,50,51,52]. In fact, past or present multicultural heritage can be an important asset when designing new tourism offers, and also for the diversification or renewal of the destination’s image [53]. The literature on this subject shows how synagogues can be considered as interesting heritage destinations for tourism.

## 3. Theoretical Foundation: Expected and Perceived Quality, Perceived Value, Satisfaction and Loyalty

The relationship between a customer and a company implies that the customer assesses various aspects of the interaction [54]. Studies have been carried out into this relationship [55,56,57] and have shown that the customer’s relationship with a company is the result of a variety of different factors. The customer’s appraisal of the company’s management of the relationship is important in the literature on marketing because there is no single factor that can evaluate the relationship independently [58]. Marketing is important for all organizations because creating and maintaining high-quality relationships with customers can have positive results for the company, such as customer cooperation, word of mouth promotion and increases in sales [58]. Therefore, following commitment theory, the relationship mediators that promote loyalty are expected quality [59], perceived quality [60], perceived value [61], satisfaction [62] and motivation [63].

Studies have shown that loyalty to a visited place is directly related to the satisfaction and opinion of the visitor [64]. Studies which analyze loyalty in tourism have mainly looked into the attitude and intention of the visitor [65]. Academic studies can be grouped into two categories due to how loyalty is examined. The first group investigates repeat purchases, that is to say, tourists returning to a destination. The second, and far more interesting, group takes loyalty to mean recommending the tourist destination to other future tourists [49,66,67]. This means that the tourist feels a connection with a destination and intends to visit it again in the future, while also recommending it to third parties [68,69,70,71,72]. It should be pointed out that there are authors who warn that a tourist’s desire for new experiences may counteract the loyalty shown to the destination [73]. Studies which investigate loyalty to cultural heritage are mainly cognitive and use structural equations to predict intentions to return to or recommend a destination [74,75].

How comfortable the tourist felt at the destination and the monument visited is one of the most important factors when deciding to return to a destination, and therefore for visitor loyalty [67,72,76]. The perceived quality is usually considered one of the most important factors in research on tourist behavior [77]. Researchers define perceived quality as the overall accumulation of the tourist’s feelings about the experience at a destination [78,79]. Tourists value their experience as positive or negative for different attributes of the monument or destination [80] Following on from this idea, the perceived value of a destination by a visitor is considered to be the most important indicator of their intentions to return to the chosen destination. It is possible that a customer does not buy a product or service because it is not considered value for money as the perceived value is not adequate for the price asked [81]. Studies by different authors explain that perceived value measures a tourist’s general assessment of their experience at a destination from the feelings they had there [82,83]. F.J. Cossío-Silva, M.A. Revilla-Camacho, and M. Vega-Vázquez (2018) obtain a realistic idea of tourist behavior by means of this variable and the results obtained can be useful for public institutions and organizations that offer tourism [84]. Customers who are aware of the value of a service or product sometimes expect particular benefits from it [85]. For this reason, perceived value is related to the usefulness of a purchase because the purchase has intrinsic benefits that satisfy the buyer’s needs [82]. A tourist who is interested in value will look for information and contemplate the idea fully before making a decision [86]. This means that perceived value affects the decisions of customers [87]. Perceived value can positively affect the loyalty of a customer [81] because customers who are aware of the value of a service make positive judgements about it, relating value to price and critically assessing all options.

## 4. Hypothesis

Loyalty is an important aspect to take into account because it is directly connected to the cost efficiency of products and services [13]. It includes variables which have an impact on the intention of tourists to visit a cultural heritage site and recommend it to others. The proposed model for this study uses four variables to measure the loyalty of Jewish tourists at the Synagogue in Córdoba: 1) The perceived quality of the attributes of the product and service. Giving a value to this variable implies the product or service has been used recently. 2) The expected quality measures that the customer expects to obtain when buying a product or service. The value given to this variable represents any past experience with or reference to a product or service [13]. 3) The perceived value refers to the advantages that a customer believes have been given by a product or service [88]. Goyhenetche (1999) states that this also implies that, with this value, the customer is also evaluating how well a product corresponds to the expected benefits [89]. The tourist experience gives value to how well what the tourist expected matches what they receive. 4) Customer satisfaction is a measure that takes into account the number of people that say they will not change their product supplier or service provider or would pay more for the product or service received.

**Figure 1:**
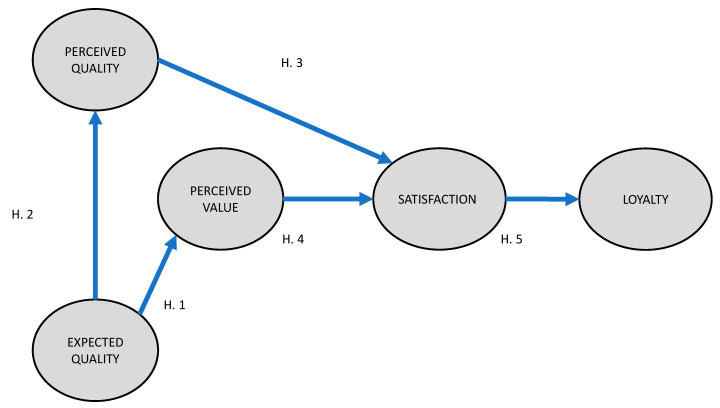
Research model.

The below hypotheses were formulated after the literature review above (see Figure 1).

**Hypothesis** **1** **(H1).** *The quality expected by the tourist of Jewish heritage significantly influences the perceived quality of the visited Jewish cultural heritage site.*

**Hypothesis** **2** **(H2).** *The quality expected by the tourist of Jewish heritage significantly influences the perceived value of the visited Jewish cultural heritage site.*

**Hypothesis** **3** **(H3).** *The quality perceived by the tourist of Jewish heritage significantly influences the satisfaction of the tourist with the visited Jewish cultural heritage site.*

**Hypothesis** **4** **(H4).** *The value perceived by the tourist of Jewish heritage significantly influences the satisfaction of the tourist with the visited Jewish cultural heritage site.*

**Hypothesis** **5** **(H5).** *The tourist’s satisfaction with the visited Jewish heritage site significantly influences their loyalty.*

## 5. Methodology

### 5.1. Data Collection and Sampling

The study was carried out in Córdoba, in the south of Spain. The synagogue in Córdoba is significant from cultural, touristic and architectural points of view [10]. This heritage site is exceptional due to the excellent conservation work carried out on its original plasterwork from Toledo, which dates from 1314 [90] and because its original architectural distribution has never been modified. It is, in fact, the most well-preserved medieval synagogue in Spain [91].

Data was collected by means of a questionnaire, which was given to tourists at the end of their visit to the synagogue. The essential requirement for participation in the data collection phase was that the tourist had to be Jewish. In order to guarantee the validity of the questionnaire, the questions used were based on those from similar previous studies [92,93]. A structured questionnaire was chosen as the most adequate data collection method for this study. In an initial phase, data collection was carried out with a pilot sample in order to collect the information while following the research aims. The information from the pilot sample was collected and its internal consistency was rigorously analyzed. A self-administered, closed questionnaire was selected as the most appropriate method of data collection, with the items being chosen from similar previous studies [94,95,96]. The validity of the survey was thus guaranteed and, lastly, two groups of experts (three researchers from the area of tourism and four professionals in tourist activities) evaluated and helped to choose the elements. The validity of the constructed items was therefore verified twice.

### 5.2. Measures and Methodology

The questionnaire had six sections. The first had questions about the demographic profile of the tourist. The following sections included questions about the tourist’s prior expectations, quality of the destination, perceived value, satisfaction and loyalty, which means their intention to return or recommend a visit to the destination/synagogue. The questions in the first four parts of the questionnaire, regarding expected quality, perceived quality, expected value, satisfaction with the destination and visitor loyalty, used a seven-point Likert scale with one being the most negative response and seven being the most positive. The team of surveyors informed the tourists about the research goal and requested their participation, voluntarily, in the study before starting the survey. Following this, the tourists responded to the questionnaire in their native language or, if this was not possible, in one that they spoke fluently. The consistency of the questionnaire was analyzed with the calculation of the Cronbach’s alpha coefficient for each construct, and for the entire questionnaire, which was composed of 79 items grouped into five question groups. The collection of the sample data was carried out by means of a questionnaire applied individually and at different times during the morning, afternoon and night. The sample was chosen exclusively from tourists with Jewish heritage who visited the monument because they have a direct relationship with the cultural heritage. For the fifth part, socio-demographic profile and travel details, closed questions were used. The Cronbach’s alpha coefficient calculated for each one of the constructs (expected quality (0,696), perceived quality (0,790), perceived value (0,682), satisfaction (0,725), loyalty (0,760)) exceeded 0.6 and, as such, it is acceptable, according to [80], who consider a scale to be acceptable if its Cronbach alpha coefficient is above 0.7. The survey was carried out in the month of April 2019, by means of a non-probability sample with intentional quotas. The number of valid questionnaires was 350 in total and, with these, a confidence level of 95% was obtained, as well as a sample error of 3.17%.

In tourism research, study variables, such as expected quality, perceived quality, the value of the tourist destination, the satisfaction of tourists and loyalty, cannot be directly measured. As such, these have to be measured by means of other observable variables. The structural equation models allow researchers to estimate relationships of multiple dependence and represent these relationships using unobserved or latent variables, bearing in mind the measurement errors in the estimation process [80]. As such, the methodology used in this study is based on structural equation models.

The research data was tabulated and analyzed using IBM SPSS 23 (IBM Corporation, Armonk, NY, USA) statistical software and the estimations of the structural equations were completed using IBM SPSS Amos 23. The initial model was designed (see the previous Figure 1) by basing it on the American Customer Satisfaction Index (ACSI) and includes the observed and latent variables. In Table 1, the unobserved and latent variables and the measurement errors of the proposed model are shown.

**Figure 2:**
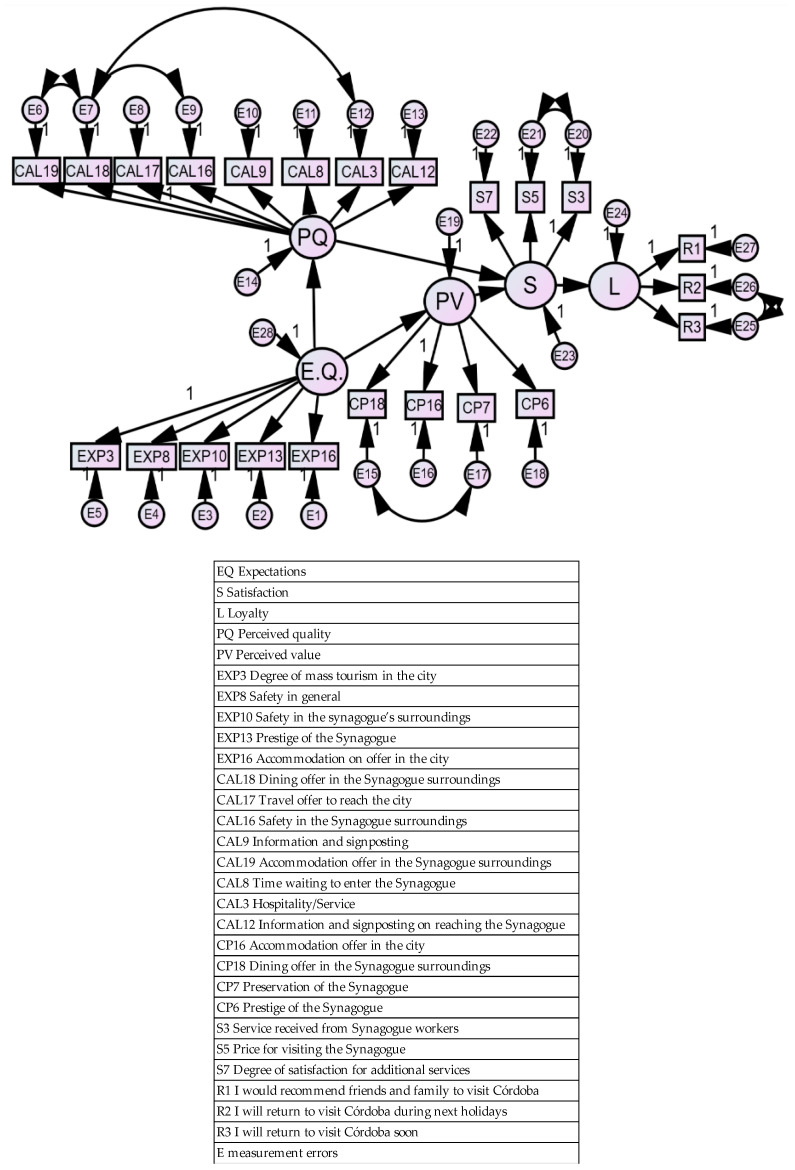
Path diagram of the proposed model.

In Figure 2, we can see the final model. The model of Maximum Likelihood (ML) was used to estimate the model. This includes the perturbation terms, the regression coefficients (these represent the relationship between exogenous and endogenous latent variables), as well as the relationship between those variables among themselves, as detailed in Table 2.

Following the recommendations in the literature, multiple indicators were used to assess the model fit [82]. The most common techniques are the chi-squared ratio regarding the degrees of freedom (CMIN/DF), the chi-squared change among alternative models, the comparative fit index (CFI), the goodness of fit index (GFI) and the root mean square error of approximation (RMSEA). The values of these goodness of fit statistics (CFI, GFI) generally vary between zero and one, with one indicating a perfect fit. Values greater than 0.9 suggest a satisfactory fit between theoretical structures and empirical data and values of 0.95 or greater suggest an optimal fit.

Lastly, besides the consideration of the model of fit, attention should be paid to the significance of the estimated parameters. These are like the regression coefficients. It would be nonsensical to consider a model that fits the data well but had fewer significant coefficients.

After validation, the valuation indices of the latent variables (ILV)—related to the perception and the expectation the tourist has of the destination as well as the perceived value, loyalty and satisfaction—were used as the weighted average, wherein every measurable variable is weighed through its coefficient with the corresponding latent variable in the estimated model:

*λ_ij_* is the standardized coefficient between the latent variable *y_i_* and the measurable variable *x_ij_*. The value *y_ik_*, calculated by every observation, is the average estimated value of each latent variable. The results obtained with this method are presented on a Likert-type scale, which, by presenting different ranges of definition, has been scaled to be expressed on a scale of one to seven.

This shall allow us to estimate a value for each latent variable, in which each one of the latent variables can be assessed quantitatively.

The descriptive analysis of the data collected in the first part of the questionnaire leads us to the conclusions that most of the tourists who travelled in the spring of 2019 were middle-aged, as 47.3% of them were between 40 and 59 years of age, with a slightly higher number of men (54.1%), while the majority of them had a higher education degree (53.3%). The sample was intentionally distributed by nationality. This way, we could assure that the distribution of national and foreign tourists would be similar to the one observed in the year 2018 (42.2% foreign tourists and 57.8% domestic tourists), so that, from the sample created by 350 valid questionnaires, 148 people surveyed were foreigners. In total, 37% were visiting the city for the first time. Most of them were staying in hotels and tourist apartments (55.1%).

In terms of satisfaction with the visit to the Synagogue of Córdoba, it is high (7.3 out of 10), as is the experience within the surroundings of the Synagogue. However, low scores are highlighted in the answers in terms of meeting the expectations of the tourists’ visit to the synagogue of Córdoba (6.57 out of 10), with the experience in the Synagogue being better scored than prior expectations. Additionally, tourists highlighted the degree of preservation, along with the prestige of this heritage site. They stated that the degree of perceived quality had surpassed their expectations.

## 6. Results

In Table 2, we show the relationship between the observed and latent variables. The structural coefficients of the normalized model have also been calculated.

**Figure 3:**
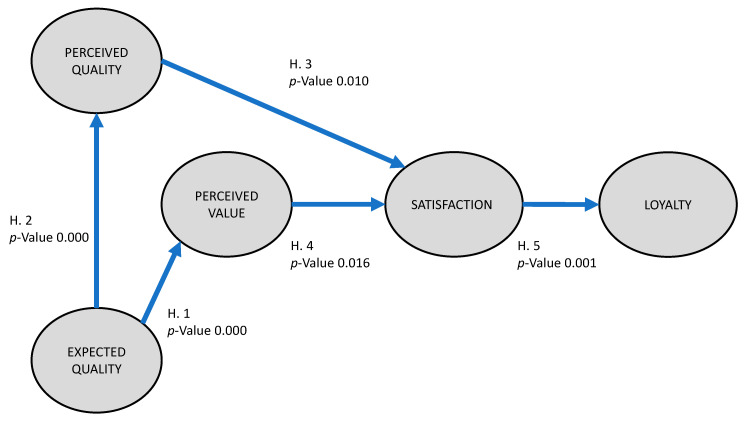
Path diagram of the proposed model with the *p* value.

In relation to latent variables, we can observe, in Table 3 and Figure 3, the intensity of the measurement of the structural coefficients. We can also see the probability limit; this validates the significance of the relationship between the constructs of the model we proposed.

To endorse the goodness of fit of the model we propose, which supports the hypothesis we formulated, the measures for absolute, incremental and parsimonious fit were calculated.

In relation to the absolute measures in Table 4, which evaluate the overall fits of the model [82], the remainder resulting from the chi-squared coefficient and the degrees of freedom (0.932) is less than 2. The Root Mean Square Error of Approximation (RMSEA) index is 0.001. Both are optimal. Some incremental fit measures, such as Adjusted Goodness of Fit Index (AGFI), are sensitive to the number of indicators and are narrowly related to the Goodness of Fit Index (GFI), with a value that indicates an acceptable fit. With respect to the parsimonious fit measures, Parsimony Goodness of Fit Indices (PNFI) and Parsimony Goodness of Fit Index (PGFI) are significantly above 0.06 when verified for the model calculated. Regarding the basis of the results obtained, the model may be said to present an appropriate goodness of fit regarding the fit indices, which, together with the significance of the model coefficients, justifies its validity and applicability.

The indices of the latent variables (IVL) were calculated for the proposed model, using the formula below:

$$IVL=\frac{\sum_{i=1}^{n}w_{i}{\bar{x}}_{i}}{\sum_{i=1}^{n}w_{i}}$$

The standardized structural coefficients of each latent variable are represented by $w_{i}$, the average of the observed variable for each latent variable by ${\bar{x}}_{i}$ and the number of observations by *n*.

This method gives standardized indices, which allow the monuments that tourists visit to be compared. The indices are calculated using the values and percentages found with the Likert scale in the questionnaire.

The results obtained for these indices in Table 5, show that the tourists who visit the synagogue are highly satisfied (78.60%) with an intermediate degree of loyalty (65.74%). An interesting point is that the perceived quality (88.09%) is higher than the expected quality (77.04%). The perceived value of the monument is very high (80.05%).

## 7. Discussion

The ACSI model was used in this study to investigate the loyalty of Jewish heritage tourists to the synagogue in Córdoba, which can play an important role in the future of the Jewish cultural experience in Spain A structural equation was created and used to study the loyalty of this type of tourists due to quality, perceived value and satisfaction with the site [108,109]. Tourists from minority heritages have special characteristics, which are important to take into account when managing heritage sites [98]. This means that tourist origins must be taken into account in academic studies and when managing heritage and making future plans for tourism at the site.

Some studies, such as [19], used 11 different interviews with key agents of the Jewish communities of Syracuse and eastern Sicily and confirmed how the management of heritage influences the political, cultural and religious identities of these minorities. Other authors such as [3], compare United Nations Educational, Scientific and Cultural Organization’s (UNESCO) Network of Creative Cities in Central and Eastern Europe, in which Jewish communities were numerous before World War II. These are Budapest (Hungary), Krakow (Poland), Prague (Czech Republic) and Kaunas (Lithuania), analyzing aspects such as creativity in wealth management using multimedia applications. This current study uses the ideas above and shows how it is essential to transmit acceptable ideas, perceptions and images of Jewish heritage, so as not to produce disagreements and conflicts within ethnic or religious communities. Problems due to the mismanagement of a heritage site are considered dangerous to the identity of the minority group, as tourism could be negatively influenced [3].

Firstly, Hypothesis 1 was confirmed and showed that the expected quality of a destination directly influences the perceived quality. The relationship between expected and perceived quality has previously been studied in publications about different economic sectors such as nursing [110] or air transport [111]. In the tourism sector, studies have been carried out into the management of tourist boats [112] and luxury hotels [113]. Most of these studies have shown how the evaluation of the tourist experience at a site is essential to gain knowledge about expected and perceived quality and value. However, this can be a difficult task as users can have different amounts of contact with the services available or with the advertising of a heritage site [114]. This study contributes to the available literature and knowledge about the area of heritage management and how to optimize this management for tourists with Jewish heritage, so that the user is not affected by disruptive factors.

Secondly, Hypothesis 2 was also confirmed and shows that the expected quality of a destination directly influences its perceived quality. A high level of expected quality is expected to have a positive effect on the value given by tourists for perceived quality [111,112]. Expected and perceived quality are the basic requirements for any successful tourist destination. Perceived value shows how quality and price influence a tourist’s feelings about the quality of a destination and means that the local community plays an important role in the feelings of tourists at a destination. A higher level of perceived value is expected to have a significantly positive effect on the satisfaction of the tourists. Quality of service gives a value to the tourist’s experience and feelings. This aspect can influence the tourist’s intention to revisit or recommend a destination or heritage site. This relationship has been studied in the tourism industry, showing that trust, quality of service and perceived value all have a positive and significant effect on customer satisfaction and that, in turn, satisfaction has a positive influence on customer loyalty [115,116]. There are also studies that confirm this relationship for the Jewish collective with products such as kosher food [117] or souvenirs [118]. The loyalty of Jewish people at Jewish heritage sites is determined by both the expected and perceived quality of the heritage site. These results are in line with other studies that analyze consumer behavior with cultural assets [119]. Factors such as the environment, cleanliness, interior decoration and ambient temperature are all part of the experience that tourists evaluate.

Thirdly, Hypothesis 3 was also confirmed and showed the direct influence that the perceived quality of a destination has on tourist satisfaction. This study therefore coincides with the results of previous research showing that the quality of the services provided and the management of the heritage site can predict customer satisfaction [120,121]. Managing employee interactions with tourists can contribute positively to improving the tourist experience. The results of the study showed that there were weak points in the management of the synagogue. It is in a privileged place, as it is located in the historical center of Córdoba, a World Heritage Site. The main weakness found was the management of tourists. Tourists tend to accumulate at certain times, while at other times the monument is empty. This increases the queues and waiting times at certain moments. The information given to tourists before their visit should also be improved, as well as the signs and indications on how to reach the Synagogue. The synagogue is situated in a privileged position, which gives the monument a great number of opportunities that the management of the site must use to transform it into a first-class tourist experience.

Hypothesis 4 was also confirmed and proves the direct influence that the perceived value of a destination has on tourist satisfaction. A higher level of perceived value is expected to have a significantly positive effect on the satisfaction of the tourists [121,122]. Cities should take advantage of the tourist resources that increase the perceived value of the destination by tourists. The Synagogue in Córdoba should therefore make Jewish heritage tourists feel that the price of the visit to this heritage site is correct. Perceived value includes various factors, both cognitive and affective, which a visitor gives a value to. These factors remind managers that heritage tourists do not only take into account the economic value of the Synagogue, but also seek values of an affective nature, such as preservation, prestige and hedonism. These findings apply to other destinations with similar heritage. These results support the findings of [123], who define customer value with three concepts: reputation for quality, value for money and prestige. The authors in [124] showed that perceived value directly and positively affects customer satisfaction.

Finally, Hypothesis 5 was confirmed, which shows the direct influence of tourist satisfaction at a destination on tourist loyalty. A higher level of satisfaction is expected to have a positive effect on the loyalty of tourists to a heritage site. This hypothesis is confirmed by previous studies on other continents [114] and those carried out in countries such as Korea [125] and various islands [126]. This study confirmed the relationship between high tourist satisfaction with the synagogue of Córdoba and the willingness of tourists to return and recommend it. This means that the loyalty of tourists with Jewish heritage to a heritage site relies on factors such as the safety of the site and surroundings, accommodation, gastronomy, transport and the management of the information given to tourists, the comfort felt and the maintenance of the site. The tourist must feel that these all make the tourist experience worth the price paid. In addition to enjoying a visit to heritage sites, tourists also appreciate special rates, offers and discounts during their visit. This result is consistent with the findings of [127,128], who discovered that price, quality and emotional value have significant effects on customer satisfaction with the tour package.

## 8. Conclusions

This study contributes to the literature on heritage management and Jewish heritage tourism in several ways. Firstly, it improves our understanding of tourism at synagogues. This can be used by policymakers to prepare and use policies for the management and hospitality at a site by including the factors found in this study. Secondly, it offers a better understanding of the relationships between heritage sites and tourists in relation to expected and perceived quality, perceived value, satisfaction and loyalty, as the hypotheses proposed in this study were strongly supported by the results.

This study has important theoretical and practical implications for the management of minority heritage sites in Spain. At a theoretical level, understanding how the ACSI model works in the cultural tourism sector can contribute to the improvement of Jewish tourism loyalty. From a practical perspective, the findings of this study corroborate the idea that wealth management can influence the feelings of minorities at a heritage site, influence the loyalty of this group and affect the number of possible positive recommendations. This means that the tourism sector should take these factors into account to improve tourist loyalty. Policymakers should explore new management methods to provide heritage tourists with a memorable experience when visiting a site. These should include adequate management of information, gastronomy, safety, prestige and preservation, which must be worth the price paid. To avoid any possible disagreements, the management of these heritage sites should pay special attention to Jewish identity and heritage considerations, including aspects such as religious, secular and institutional orientation, in addition to touristic commodification of a site. Another possible area of conflict could be the non-Jewish interest in Jewish heritage tourism, because public authorities, private operators and tourists themselves may not have any special interest in heritage in terms of interaction or cultural exchange with the Jewish community. This synagogue is a cultural heritage site in Córdoba of Jewish origin. However, most of those interested in advertising it are not of Jewish origin, which makes the adequate promotion of the heritage site difficult. However, the local tourism board has a new system for the promotion of this type of niche tourism [53,129], which aims to differentiate, diversify and enrich tourism in the city [129] by endorsing the concept of a multicultural and cosmopolitan city. The question is whether this can be carried out without highlighting and exacerbating conflicting views and values within the minority itself, i.e., without creating discord in the community [130]. Public administration and private businesses which are responsible for the management of the synagogue must work together to improve the tourist experience [131,132,133].

This research work has potential limitations, the first of which is the sample used. The data was only obtained from tourists at the synagogue of Córdoba, which could indicate that the collected data is indicative of only one particular tourist site. This can be improved by using a longitudinal study to register the feelings of tourists over time, which will allow for a more accurate measurement of the relationships between the variables. Additionally, measuring loyalty from future behavioral intentions is another limitation of the study. Although this method has been used in various studies, it is still a subjective measure of behavior and does not always correspond to actual behavior [134].

## Figures and Tables

**Table 1:** Scales used.

Reference	Dimension	Indicators
[77,97,98,99]	Expectations (EQ)	(EXP3) Degree of mass tourism in the city (EXP8) Safety in general (EXP10 Safety in the synagogue’s surroundings (EXP13 Prestige of the Synagogue (EXP16) Accommodation on offer in the city
[62,100,101]	Satisfaction (S)	(S3) Service received from Synagogue workers (S5) Price for visiting the Synagogue (S7) Degree of satisfaction for additional services
[67,72,76,81,102,103]	Loyalty (L)	(R1) I would recommend friends and family to visit Córdoba (R2) I will return to visit Córdoba during next holidays (R3) I will return to visit Córdoba soon
[61,79,80,104,105,106,107]	Perceived quality (PQ)	(CAL18) Dining offer in the Synagogue surroundings (CAL17) Travel offer to reach the city (CAL16) Safety in the Synagogue surroundings (CAL9) Information and signposting (CAL19) Accommodation offer in the Synagogue surroundings (CAL8) Time waiting to enter the Synagogue (CAL3) Hospitality/Service (CAL12) Information and signposting on reaching the Synagogue
[61,88,101]	Perceived value (PV)	(CP16) Accommodation offer in the city (CP18) Dining offer in the Synagogue surroundings (CP7) Preservation of the Synagogue (CP6) Prestige of the Synagogue

**Table 2:** Standardized structural coefficients of the observed variables regarding the latent variables.

Latent Variable	Observed Variable	Standardized Coefficient
Expected Quality	EXP3 Degree of mass tourism in the city	0.333
	EXP8 Safety in general	0.421
	EXP10 Safety in the synagogue’s surroundings	0.564
	EXP13 Prestige of the Synagogue	0.633
	EXP16 Accommodation on offer in the city	0.680
Perceived Quality	CAL18 Dining offer in the Synagogue surroundings	0.657
	CAL17 Travel offer to reach the city	0.6
	CAL16 Safety in the Synagogue surroundings	0.476
	CAL9 Information and signposting	0.612
	CAL19 Accommodation offer in the Synagogue surroundings	0.675
	CAL8 Time waiting to enter the Synagogue	0.614
	CAL3 Hospitality/Service	0.423
	CAL12 Information and signposting on reaching the Synagogue	0.328
Perceived Value	CP16 Accommodation offer in the city	0.239
	CP18 Dining offer in the Synagogue surroundings	0.671
	CP7 Preservation of the Synagogue	0.548
	CP6 Prestige of the Synagogue	0.639
Satisfaction	S3 Service received from Synagogue workers	0.585
	S5 Price for visiting the Synagogue	0.587
	S7 Degree of satisfaction for additional services	0.639
Loyalty	R1 I would recommend friends and family to visit Córdoba	0.513
	R2 I will return to visit Córdoba during next holidays	0.646
	R3 I will return to visit Córdoba soon	0.657

**Table 3:** Standardized coefficients and *p*-values that support the hypothesis.

Hypothesis	Latent Variable	Latent Variable	Standardized Coefficient	*p* Value
H1	Expected quality	Perceived value	0.759	0.000
H2	Expected quality	Perceived quality	1.015	0.000
H3	Perceived quality	Satisfaction	0.468	0.010
H4	Perceived value	Satisfaction	0.451	0.016
H5	Satisfaction	Loyalty	0.653	0.001

**Table 4:** The goodness of fit model.

Fit of the Model	Value
**Measures of Absolute Fit**	
Chi-square/DF	0.932
GFI	0.896
RMSEA	0.001
RMR	0.069
**Measures of Incremental Fit**	
AGFI	0.858
**Measures of Parsimonious Fit**	
PNFI	0.685
PCFI	0.798

**Table 5:** The indices of the latent variables (IVL).

Latent Variable Index (IVL)	Likert Scale	Percentage
Expected Quality Index	5392	77.04%
Perceived Quality Index	6166	88.09%
Perceived Value Index	5603	80.05%
Satisfaction Index	5502	78.60%
Loyalty Index	4602	65.74%

## References

[1] Koren-Lawrence N., Collins-Kreiner N. (2019). Visitors with their ‘Backs to the archaeology’: Religious tourism and archaeology. J. Herit. Tour..

[2] Corpas N., Castillo A. (2019). Tourism 3.0 and archaeology: Approaching tourists’ generated-content of World Heritage sites. Pasos Rev. Tur. Patrim. Cult..

[3] Gaižutytė-Filipavičienė Ž. (2020). Jewish heritage in the creative cities of Central and Eastern Europe: Tourism, technologies and prosthetic memory. Creat. Stud..

[4] Li T.E., McKercher B., Chan E.T.H. (2019). Towards a conceptual framework for diaspora tourism. Curr. Issues Tour..

[5] Kidron C.A. (2013). Being there together: Dark family tourism and the emotive experience of co-presence in the holocaust past. Ann. Tour. Res..

[6] Kidron C.A. (2015). Survivor Family Memory Work at Sites of Holocaust Remembrance: Institutional Enlistment or Family Agency?. Hist. Mem..

[7] Kandiyoti D. (2019). Modern Spain and the Sephardim: Legitimizing identities (Lexington studies in modern Jewish history, historiography, and memory). Jew. Cult. Hist..

[8] Kaplan G.B. (2019). Jewish Poetry and Cultural Coexistence in Late Medieval Spain.

[9] De San Eugenio J., Ginesta X., Compte-Pujol M., Frigola-Reig J. (2019). Building a Place Brand on Local Assets: The Case of the Pla de l’Estany District and Its Rebranding. Sustainability.

[10] Centella R. (1992). La Judería de Córdoba. El Almendro.

[11] Observatorio de Turismo de Córdoba. https://www.turismodecordoba.org/boletines-trimestrales-observatorio-turistico.

[12] Corsale A., Vuytsyk O. (2018). Jewish heritage tourism between memories and strategies. Different approaches from Lviv, Ukraine. Curr. Issues Tour..

[13] Fornell C., Johnson M.D., Anderson E.W., Cha J., Bryant B.E. (1996). The American Customer Satisfaction Index: Nature, purpose, and findings. J. Mark..

[14] Ahsan A., Pratiwi Y., Nursalam N., Efendi F. (2019). Model Development of Nursing Service Loyalty. Indianj. Public Health Res. Dev..

[15] Hult G.T.M., Sharma P.N., Morgeson F.V., Zhang Y. (2019). Antecedents and consequences of customer satisfaction: Do they differ across online and offline purchases?. J. Retail..

[16] Hoang D.P. (2019). The central role of customer dialogue and trust in gaining bank loyalty: An extended SWICS model. Int. J. Bank Mark..

[17] Bezerra G.C., Gomes C.F. (2019). Determinants of passenger loyalty in multi-airport regions: Implications for tourism destination. Tour. Manag. Perspect..

[18] Gogoi B.J. (2020). Service quality measures: How it impacts customer satisfaction and loyalty. Int. J. Manag..

[19] Corsale A., Krakover S. (2019). Cultural tourism between local and transnational identities: Jewish heritage in Syracuse, Italy. Tour. Geogr..

[20] Blankenship A.M. (2019). Jewish Tourism in Berlin and Germany’s Public Repentance for the Holocaust. Acad. Tour. Innov. J..

[21] Lützen K.C. (2019). To Walk in the Footsteps of Your Ancestors: Roots Tourism in Yiddishland. New Perspectives on Jewish Cultural History: Boundaries, Experiences, and Sensemaking.

[22] Chołuj A., Gawrecki K. (2019). Jewish quarter in Medieval Lorca (south-eastern Spain). Tur. Kult..

[23] Fu X. (2019). Existential authenticity and destination loyalty: Evidence from heritage tourists. J. Destin. Mark. Manag..

[24] Ramires A., Brandao F., Sousa A.C. (2018). Motivation-based cluster analysis of international tourists visiting a World Heritage City: The case of Porto, Portugal. J. Destin. Mark. Manag..

[25] Kim M., Thapa B. (2018). Perceived value and flow experience: Application in a nature-based tourism context. J. Destin. Mark. Manag..

[26] Prayag G., Hosany S., Odeh K. (2013). The role of tourists’ emotional experiences and satisfaction in understanding behavioral intentions. J. Destin. Mark. Manag..

[27] Meng B., Han H. (2018). Working-holiday tourism attributes and satisfaction in forming word-of-mouth and revisit intentions: Impact of quantity and quality of intergroup contact. J. Destin. Mark. Manag..

[28] Basu P. (2004). My own island home: The Orkney homecoming. J. Mater. Cult..

[29] Basu P. (2005). Macpherson Country: Genealogical identities, spatial histories and the Scottish diasporic clanscape. Cult. Geogr..

[30] McCain G., Ray N.M. (2003). Legacy tourism: The search for personal meaning in heritage travel. Tour. Manag..

[31] Meethan K., Colles T., Timothi D.J. (2004). To stand in the shoes of my ancestors? Tourism and genealogy. Tourism, Diasporas and Space.

[32] Alexander M., Bryce D., Murdy S. (2017). Delivering the past: Providing personalized ancestral tourism experiences. J. Travel Res..

[33] Lee W., Chhabra D. (2015). Heritage hotels and historic lodging: Perspectives on experiential marketing and sustainable culture. J. Herit. Tour..

[34] Cole T., Popescu D., Schult T. (2015). Holocaust Tourism: The Strange yet Familiar/the Familiar yet Strange. Revisiting Holocaust Representation in the Post-Witness Era. The Holocaust and Its Contexts.

[35] Bousfield J., Salter M. (2009). The Rough Guide to Poland.

[36] Ojalvo R., Akpınar İ. (2017). A monument for Turkish hospitality: The staging of Ahrida Synagogue. J. Archit..

[37] Boussetta M. (2018). Reducing barriers how the Jews of Djerba are using tourism to assert their place in the modern nation state of Tunisia. J. Northafrican Stud..

[38] Corsale A. (2017). Jewish heritage tourism in Bucharest: Reality and visions. Geogr. J..

[39] Riu Cabanas R. (1892). Aljamahebrea de Solsona.

[40] González F. (1885). Tres Manuscritosrabínicos.

[41] Romero R. (1878). La sinagoga de Córdoba, Hoy Ermitadedicada al Culto Bajo la Advocación de San Crispín.

[42] Rodríguez J.A.E., Carrillo J.G., Ballesteros J.A.G. (2017). La sinagogabajomedieval de Lorca: Balance de 15 años de investigaciones y nuevasperspectivas. Miscelánea Estud. Árabes Hebr. Sección Hebr..

[43] Bravo B.M.E. (2002). Restauración y rehabilitación de un edificiohistóricoolvidado: La Sinagoga de Valencia de Alcántara (Cáceres). Norba Rev. Arte.

[44] Prat J.M., Cànoves G. (2016). La revalorizaciónturística del patrimoniojudíoenEspaña. Sus barrios y su vino. Investig. Turísticas.

[45] Forga J.M.P. (2019). El enoturismo Casher enEspaña: Un nuevo productoturístico y cultural. Rotur Rev. Ocio Tur..

[46] Ashworth G., Page S.J. (2011). Urban tourism research: Recent progress and current paradoxes. Tour. Manag..

[47] Timothy D.J., Boyd S.W. (2003). Heritage Tourism.

[48] Ashworth G.J., Graham B., Tunbridge J.E. (2007). Pluralizing pasts. Heritage, identity and place. Multicultural Societies.

[49] Castro C.B., Armario E.M., Ruiz D.M. (2007). The influence of market heterogeneity on the relationship between a destination’s image and tourists’ future behaviour. Tour. Manag..

[50] Hoffman L.M. (2003). The marketing of diversity in the inner city: Tourism and regulation in Harlem. Int. J. Urban. Reg. Res..

[51] Krakover S., Vargas-Sánchez A. (2013). Generation of a tourism product: Jewish heritage tourism in Spain. Enlightening Tourism.

[52] Ma M., Hassink R. (2013). An evolutionary perspective on tourism area development. Ann. Tour. Res..

[53] Dinis A., Krakover S. (2016). Niche tourism in small peripheral towns: The case of Jewish heritage in Belmonte, Portugal. Tour. Plan. Dev..

[54] Jap S.D., Manolis C., Weitz B.A. (1999). Relationship quality and buyer–Seller interactions in channels of distribution. J. Bus. Res..

[55] Grégoire Y., Tripp T.M., Legoux R. (2009). When customer love turns into lasting hate: The effects of relationship strength and time on customer revenge and avoidance. J. Mark..

[56] Przybytniowski J.W. (2020). Methods of service quality assessment in managing the property insurance market. Bus. Public Adm. Stud..

[57] Johnson J.L. (1999). Strategic integration in industrial distribution channels: Managing the interfirm relationship as a strategic asset. J. Acad. Mark. Sci..

[58] Palmatier R.W., Dant R.P., Grewal D., Evans K.R. (2006). Factors influencing the effectiveness of relationship marketing: A meta-analysis. J. Mark..

[59] Ferri Sanz M., DuráFerrandis E., Garcés Ferrer J. (2019). Service quality scales and tourists with special needs: A systematic review. Sustainability.

[60] Suhartanto D., Brien A., Primiana I., Wibisono N., Triyuni N.N. (2020). Tourist loyalty in creative tourism: The role of experience quality, value, satisfaction, and motivation. Curr. Issues Tour..

[61] Mohseni S., Jayashree S., Rezaei S., Kasim A., Okumus F. (2018). Attracting tourists to travel companies’ websites: The structural relationship between website brand, personal value, shopping experience, perceived risk and purchase intention. Curr. Issues Tour..

[62] Alrwajfah M.M., Almeida-García F., Cortés-Macías R. (2019). Residents’ perceptions and satisfaction toward tourism development: A case study of Petra Region, Jordan. Sustainability.

[63] Kim M.J., Hall C.M. (2019). A hedonic motivation model in virtual reality tourism: Comparing visitors and non-visitors. Int. J. Inf. Manag..

[64] Paul J., Modi A., Patel J. (2016). Predicting green product consumption using theory of planned behavior and reasoned action. J. Retail. Consum. Serv..

[65] Buhalis D., López E.P., Martinez-Gonzalez J.A. (2020). Influence of young consumers’ external and internal variables on their e-loyalty to tourism sites. J. Destin. Mark. Manag..

[66] Chen C.F., Tsai D. (2007). How destination image and evaluative factors affect behaviouralintentions?. Tour. Manag..

[67] Sato S., Kim H., Buning R.J., Harada M. (2018). Adventure tourism motivation and destination loyalty: A comparison of decision and non-decision makers. J. Destin. Mark. Manag..

[68] Baker D.A., Crompton J.L. (2000). Quality, satisfaction and behavioural intentions. Ann. Tour. Res..

[69] Kozak M. (2001). Repeaters’ Behavior at Two Distinct Destinations. Ann. Tour. Res..

[70] Kozak M., Rimmington M. (2000). Tourist Satisfaction with Mallorca, Spain, as an Off-Season Holiday Destination. J. Travel Res..

[71] Yoon Y., Uysal M. (2005). An Examination of the Effects of Motivation and Satisfaction on Destination Loyalty: A Structural Model. Tour. Manag..

[72] Chi C.G.Q., Qu H. (2008). Examining the Structural Relationships of Destination Image, Tourist Satisfaction and Destination Loyalty: An integrated approach. Tour. Manag..

[73] Almeida-Santana A., Moreno-Gil S. (2018). Understanding tourism loyalty: Horizontal vs. destination loyalty. Tour. Manag..

[74] Kang J.W., Lee N. (2018). The impact of restaurant patrons’ flow experience on SNS satisfaction and offline purchase intentions. Int. J. Contemp. Hosp. Manag..

[75] Yu T.W., Chen T. (2018). Online travel insurance purchase intention: A transaction cost perspective. J. Travel Tour. Mark..

[76] Yuksel A., Yuksel F., Bilim Y. (2009). Destination attachment: Effects on customer satisfaction and cognitive, affective and conative loyalty. Tour. Manag..

[77] Wu H.-C., Cheng C.-C., Ai C.-H. (2017). A study of experiential quality, equity, happiness, rural image, experiential satisfaction, and behavioral intentions for the rural tourism industry in China. Int. J. Hosp. Tour..

[78] Jin N., Lee S., Lee H. (2015). The effect of experience quality on perceived value, satisfaction, image and behavioral intention of water park patrons: New versus repeat visitors. Int. J. Tour. Res..

[79] Loureiro S.M.C., González F.J.M. (2008). The importance of quality, satisfaction, trust, and image in relation to rural tourist loyalty. J. Travel Tour. Mark..

[80] Nair V., Hussain K., Chiun L.M., Ragavan M.N.A., Rajaratnam S.D., Sharif S.P., Munikrishnan U.T. (2015). Destination quality and tourists’ behavioural intentions: Rural tourist destinations in Malaysia. Worldw. Hosp. Tour. Themes.

[81] Lichtenstein D.R., Netemeyer R.G., Burton S. (1990). Distinguishing coupon proneness from value consciousness: An acquisition-transaction utility theory perspective. J. Mark..

[82] Chang L.-L., Backman K.F., Huang Y.C. (2014). Creative tourism: A preliminary examination of creative tourists’motivation, experience, perceived value and revisit intention. Int. J. Cult. Tour. Res..

[83] Chen C.F., Chen F.S. (2018). Antecedents and consequences of perceived coolness for generation Y in the context of creative tourism—A case study of the pier 2 art Center in Taiwan. Tour. Manag..

[84] Cossío-Silva F.J., Revilla-Camacho M.-A., Vega-Vázquez M. (2018). The tourist loyalty index: A new indicator for measuring tourist destination loyalty?. J. Innov. Knowl. J. Innov. Knowl..

[85] Bao Y., Mandrik C.A. (2004). Discerning store brand users from value consciousness consumers: The role of prestige sensitivity and need for cognition. Acr N. Am. Adv..

[86] Pillai K.G., Kumar V. (2012). Differential effects of value consciousness and coupon proneness on consumers’ persuasion knowledge of pricing tactics. J. Retail. Pricing Glob. Marketpl..

[87] Delgado-Ballester E., Hernandez-Espallardo M., Rodriguez-Orejuela A. (2014). Store image influences in consumers’ perceptions of store brands: The moderating role of value consciousness. Eur. J. Mark..

[88] Nilson T.H. (1992). Value-Added Marketing: Marketing Management for Superior Results.

[89] Goyhenetche M. (1999). Créer de la Valeur Pour le Client: Le Marketing de la Valeur.

[90] JordanoBarbudo M.A. (2011). La Sinagoga de Córdoba y Las Yeseríasmudeéjaresenla Baja Edad Media.

[91] Peláez J. (1990). La Sinagoga.

[92] Kock N. (2015). Common method bias in PLS-SEM: A full collinearity assessment approach. Int. J. E-Collab..

[93] Farooq M.S., Salam M., Fayolle A., Jaafar N., Ayupp K. (2018). Impact of service quality on customer satisfaction in Malaysia airlines: A PLS-SEM approach. J. Air Transp. Manag..

[94] Kozak M. (2002). Comparative analysis of tourist motivations by nationality and destinations. Tour. Manag..

[95] Anderson W. (2012). Analysis of ‘all-inclusive’ tourism mode in the Balearic Islands, Tourism: An International Multidisciplinary. J. Tour..

[96] Özdemir B., Çizel B., Çizel R.B. (2012). Satisfaction with all-inclusive tourism resorts: The effects of satisfaction with destination and destination loyalty. Int. J. Hosp. Tour. Adm..

[97] Zehrer A., Smeral E., Hallmann K. (2017). Destination competitiveness—A comparison of subjective and objective indicators for winter sports areas. J. Travel Res..

[98] Kiatkawsin K., & Han H. (2017). Young travelers’ intention to behave pro-environmentally: Merging the value-belief-norm theory and the expectancy theory. Tour. Manag..

[99] Hallak R., Assaker G., El-Haddad R. (2018). Re-examining the relationships among perceived quality, value, satisfaction, and destination loyalty: A higher-order structural model. J. Vacat. Mark..

[100] Senarath S.M.U.C., Ranasinghe J.P.R.C. Destination Image and Destination Loyalty: Comparison between Asia Pacific and European Tourists. Proceedings of the International Research Conference of UWU.

[101] Kim W.G., & Park S.A. (2017). Social media review rating versus traditional customer satisfaction. Int. J. Contemp. Hosp. Manag..

[102] Kang J.W., & Namkung Y. (2018). The effect of corporate social responsibility on brand equity and the moderating role of ethical consumerism: The case of Starbucks. J. Hosp. Tour. Res..

[103] Wang K., Chen Y.J., Pan H.C. Determinants of Brand Loyalty in Online Brand Communities: The View of Place Attachment. Proceedings of the 5th Multidisciplinary International Social Networks Conference.

[104] Jin N., Line N.D., & Merkebu J. (2016). The impact of brand prestige on trust, perceived risk, satisfaction, and loyalty in upscale restaurants. J. Hosp. Mark. Manag..

[105] Carneiro J., Saraiva P., Conceição L., Santos R., Marreiros G., & Novais P. (2019). Predicting satisfaction: Perceived decision quality by decision-makers in web-based group decision support systems. Neurocomputing.

[106] Nedelea A.M., Ali M.B., Alamgir M. (2017). Factors behind destination loyalty-a study on tourism destination. Rev. De Tur. -Stud. Si Cercet. Tur..

[107] Dieck M.C., & Jung T.H. (2017). Value of augmented reality at cultural heritage sites: A stakeholder approach. J. Destin. Mark. Manag..

[108] Astor A., Burchardt M., Griera M. (2019). Polarization and the Limits of Politicization: Cordoba’s Mosque-Cathedral and the Politics of Cultural Heritage. Qual. Sociol..

[109] Ashworth G.J., Graham B.J., Tunbridge J.E. (2007). Pluralising Pasts: Heritage, Identity and Place in Multicultural Societies.

[110] Jarrar M.T., Minai M.S., Al-Bsheish M., Meri A., Jaber M. (2019). Hospital nurse shift length, patient-centered care, and the perceived quality and patient safety. Int. J. Health Plan. Manag..

[111] Chen L., Li Y.Q., Liu C.H. (2019). How airline service quality determines the quantity of repurchase intention-Mediate and moderate effects of brand quality and perceived value. J. Air Transp. Manag..

[112] Mehran J., Olya H.G. (2020). Canal boat tourism: Application of complexity theory. J. Retail. Consum. Serv..

[113] Varsanis K., Belias D., Kakkos N., Chondrogiannis M., Rossidis I., Mantas C. (2019). The Relationship between Service Quality and Customer Satisfaction on Luxurious Hotels so to Produce Error-Free Service. Strateg. Innov. Mark. Tour..

[114] Germann R., Kurth L., Matthiesen S. (2020). Disruptive factors in the evaluation of perceived quality aspects—Consideration of the brand influence. Int. J. Ind. Ergon..

[115] Rico M.T., Wahyoedi S., Purnama E.D. The Effects of Trust, Service Quality and Perceived Value on Satisfaction and Their Impact on Loyalty. Proceedings of the 7th International Conferenceon Entrepreneurship and Business Management.

[116] Dedeoğlu B.B. (2019). Shaping tourists’ destination quality perception and loyalty through destination country image: The importance of involvement and perceived value. Tour. Manag. Perspect..

[117] Jeong E., Yang Q., Lee S., Bosselman R. (2019). Factors for non-Jewish consumers’ kosher food choice: An investigation of the food quality perception. J. Foodserv. Bus. Res..

[118] Shtudiner Z., Klein G., Zwilling M., Kantor J. (2019). The value of souvenirs: Endowment effect and religion. Ann. Tour. Res..

[119] Alrawadieh Z., Prayag G., Alrawadieh Z., Alsalameen M. (2019). Self-identification with a heritage tourism site, visitors’ engagement and destination loyalty: The mediating effects of overall satisfaction. Serv. Ind. J..

[120] Kim K.H., Park D.B. (2017). Relationships among perceived value, satisfaction, and loyalty: Community-based ecotourism in Korea. J. Travel Tour..

[121] Hidalgo-Fernández A., Hernández-Rojas R., del Río J., Antonio J., Casas-Rosal J.C. (2019). Tourist Motivations and Satisfaction in the Archaeological Ensemble of Madinat Al-Zahra. Sustainability.

[122] PandžaBajs I. (2015). Tourist perceived value, relationship to satisfaction, and behavioral intentions: The example of the Croatian tourist destination Dubrovnik. J. Travel Res..

[123] Nasution H.N., Mavondo F.T. (2008). Customer value in the hotel industry: What managers believe they deliver and what customer experience. Int. J. Hosp. Manag..

[124] Wu C.H.J., Liang R.D. (2009). Effect of experiential value on customer satisfaction with service encounters in luxury-hotel restaurants. Int. J. Hosp. Manag..

[125] Senarath U., Ranasinghe J.P.R.C. (2019). Destination Image and Loyalty: Comparison between Asia Pacific and European Tourists in Sri Lanka. J. Manag. Tour. Res. (Jmtr).

[126] Sangpikul A. (2018). The effects of travel experience dimensions on tourist satisfaction and destination loyalty: The case of an island destination. Int. J. Cult. Tour. Hosp. Res..

[127] Eid R. (2015). Integrating Muslim customer perceived value, satisfaction, loyalty and retention in thetourismindustry: Anempiricalstudy. Int. J. Tour..

[128] Eid R., El-Gohary H. (2015). The role of Islamic religiosity on the relationship between perceived value and tourist satisfaction. Tour. Manag..

[129] Krakover S. (2016). A heritage site development model: Jewish heritage product formation in south-central Europe. J. Herit. Tour..

[130] Tunbridge J.E., Ashworth G.J. (1996). Dissonant Heritage: The Management of the Past as a Resource in Conflict.

[131] Bosque I.A.R., Martín H.S., Collado J. (2006). The role of expectations in the consumer satisfaction formation process: Empirical evidence in the travel agency sector. Tour. Manag..

[132] Fornell C., Mithas S., Morgeson F.V., Krishnan M.S. (2006). Customer satisfaction and stock prices: High returns, low risk. J. Mark..

[133] Song H., Li G., van der Veen R., Li G., Chen J.L. (2012). The Hong Kong tourist satisfaction index. Ann. Tour. Res..

[134] Bodet G. (2012). Loyalty in sport participation services: An examination of the mediating role of psychological commitment. J. Sport Manag..

